# Stress During Lactation: A Hidden Link to Offspring Bone Health

**DOI:** 10.1007/s00223-025-01378-6

**Published:** 2025-05-30

**Authors:** Ranjitha Chandrashekar, Bharath K. Mulakala, Manoj Gurung, Geetanjali Venna, Jolene R. Rearick, Brenda Onyekweli, Meghan L. Ruebel, Jasmine Dada-fox, Jasmina A. Zeledon, Rachelanne Talatala, Kayleigh Rodriguez, Laura R. Osborn, Mary Grace Bishop, Brenda Smith, Kimberly E. Stephens, Edralin A. Lucas, Laxmi Yeruva

**Affiliations:** 1 Nutritional Sciences Department, Oklahoma State University, Stillwater, OK 74078 USA; 2 Microbiome and Metabolism Research Unit (MMRU), USDA-ARS, Arkansas Children’s Nutrition Center, Southeast Area, 15 Children’s Way, Little Rock, AR 72202 USA; 3 Texas A & M, IHA, College Station, TX USA; 4 University of Arkansas for Medical Sciences, Little Rock, AR 72202 USA; 5 Department of Obstetrics and Gynecology, & Indiana Center for Musculoskeletal Health, Indiana School of Medicine, Indianapolis, IN USA

**Keywords:** Early life stress, Bone health, Bone mineral density, Gene expression

## Abstract

Early-life resource limitation is one factor that could have a major impact on child health and development. Thus, using a rat model of limited bedding and nesting (LBN), we investigated the postnatal bone development, mineralization, and microarchitecture. Pregnant Sprague–Dawley rats were subjected to a LBN model to induce chronic early-life stress (CES), while a control group was maintained under standard conditions. The offspring were assessed at postnatal day (PND) 10, 21, and 35. Tibial length was measured, and tibial and lumbar vertebral bone mineral density (BMD), content (BMC), and area (BMA) were assessed using dual-energy X-ray absorptiometry (DXA). Bone microarchitecture was examined using microcomputed tomography (μCT). Changes in gene expression from the lumbar vertebrae were analyzed by transcriptome. At PND 10, there were no significant differences in BMD and BMC between the treatment groups, but tibial length was significantly decreased by CES. By PND 21, tibial BMC and BMA were significantly reduced in the CES group, indicating impaired bone mineral accumulation. At PND 35, tibial length remained significantly reduced by CES, while BMD and BMC differences were less affected. Vertebral BMA and BMC were reduced by CES. μCT analysis of tibial cortical bone showed significant changes in cortical thickness and bone volume at PND 10 and 21, respectively. For the lumbar vertebrae, μCT data indicated significant increases in the degree of anisotropy and structural model index at PND 21 and 35, respectively. Transcriptome analyses revealed significant differential expression of genes involved in immune response, cellular repair, and stress adaptation at PND 21 but not at PND 10 and PND 35. CES significantly disrupts BMC, BMD, length, and microarchitecture differently at various stages of postnatal development. Transcriptome analyses suggest that these changes are mediated by alterations in gene expression related to immune function and cellular repair. Future research should focus on tracking the longitudinal impacts of CES on bone health from infancy into adulthood, and exploring nutritional interventions, stress reduction programs, and molecular studies that can mitigate the negative effects of CES on bone.

**Supplementary Information:**

The online version contains supplementary material available at 10.1007/s00223-025-01378-6.

## Introduction

In the United States, approximately, 65% of individuals experience adverse early-life events. In 2007 alone, nearly 3.5 million children were reported to have undergone significant stress. This number has since doubled, with an estimated 6 to 7 million children exposed to stressful events annually between 2016 and 2019 [1, 2]. Stress has a significant impact on physical, mental, and social well-being of an individual [3]. Several factors, such as trauma, abuse, separation, poor maternal health, limited resources, nutrition, environmental and social deprivation, contribute to early-life stress (ELS). ELS, one of the major stresses experienced during childhood, can be acute or chronic and can occur as early as the prenatal period, during *in-utero* development, postnatal period within the first few months of life, and throughout childhood. The prenatal and early infancy period are critical stages of growth and development of a child [4] and especially for bone growth and mineralization and overall bone health [5]. Thus, exposure to any form of stress during these critical periods of development can have long-term health impact such as increased risk of bone fractures throughout their life and into adulthood [6, 7].

Studies have suggested that mental stress and depression, in addition to physical stress, could be a predisposing factor to bone abnormalities, including low bone mineral density (BMD) and fractures [8–13]. Early postnatal period is a rapid bone growth and mineralization stage characterized by rapid elongation and change in size and shape fostered by calcium input from the intestine, renal reabsorption, and calcium store [14]. Adaptation and adjustment to the external environment after birth are needed for optimal bone development, including architecture, bone mass, BMD, and strength. In newborns, during the first 6 months of age, while the endocortical BMD decreases, the bone marrow cavity expands, and bone elongates [15–17]. Although the endocortical bone tissue is reduced, its distribution is shifted to the periosteal surface to compensate for the growing bone length, and in fact, an increase in bone length occurs before bone mass increase [15, 18]. Hence, early childhood is when bone remodeling and adaptation to environmental changes continuously occur. The disruption in this physiology of early-life bone development can contribute to an increased risk of bone fractures and bone fragility into adulthood [19–21]. These reports suggest that stress leads to impairment in bone health. However, studies on offspring experiencing early-life resource limitations during lactation and its impact on postnatal bone development are limited.

Several animal models of ELS have been developed to study childhood adversity and its effects on health outcomes. Prenatal ELS models include maternal restraint, unpredictable stress via foot shocks, and forced swimming during gestation [22, 23]. Postnatal ELS models include daily maternal separation [24–27], chronic early-life stress (CES) introduced through male intruder exposure during early postnatal days [28–31], and limited bedding and nesting (LBN) conditions. The LBN model involves reducing bedding materials and employing mesh platforms to induce fragmented, unpredictable maternal care, and resource scarcity, simulating aspects of neglect and abuse [32–34]. Although animal stress models cannot fully replicate the psychological and social complexities of human stress, physiological responses such as activation of the hypothalamic–pituitary–adrenal (HPA) axis and altered bone metabolism are conserved across species. Limited research has evaluated the effects of the LBN model on bone health during critical developmental windows.

Here, we aim to evaluate the effects of an impoverished environment during the early postnatal period on bone health using the CES rodent model (Molet et al.). This model employs a limited nesting and bedding paradigm to provoke stress in the dam and/or her pups from postnatal day (PND) 2 through 10. While the relationship between the early-life environment and adult bone health is multifaceted and complex, the CES model provides a controlled scenario in which the caregiver is present, but resources are depleted throughout a critical window of bone development. To address the existing knowledge gap, we measured gene expression profiles of the lumbar vertebrae in male and female offspring at PND 10, 21, and 35 and comprehensively evaluated their bone health parameters. Our study provides insights into how resource depletion during early life alters gene expression in bone tissues and affects bone health.

## Materials and Methods

### Study Design

**Fig. 1:**
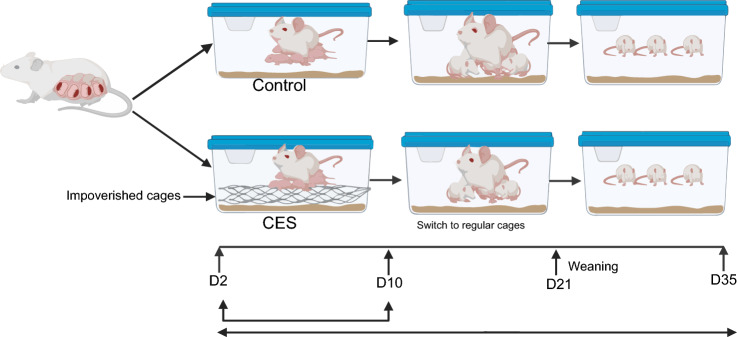
Limited bedding and nesting model of stress. To induce chronic early-life stress (CES) in the rat model, CES cages were provided limited bedding and nesting from postnatal days 2 to 10 and bedding separated with mesh wire, while standard group had access to bedding and nesting material. On day 10, CES dams were transferred to a standard cage with access to bedding and nesting until day 21. The offspring were weaned and housed in a standard cage until day 35. STD—control

Animal experiments were performed at Arkansas Children’s Research Institute as approved by the rats were housed in 12-h light/dark conditions with ad libitum access to a standard rodent chow (Lab diets, Prolab RMH1800, St. Louis. MO) and water. Timed pregnant Sprague–Dawley rats (Charles River, Wilmington, MA) were delivered to the vivarium on gestational day 18. Dams delivered pups without intervention on PND 0. On PND 2, the dams were randomly transferred to cages containing a standard plexiglass cage with ample sawdust and 3 paper towels to use as nesting material (STD), or an impoverished cage (CES) which had limited sawdust which was inaccessible to the dam by a wire mesh platform which sat 2 cm above the cage floor and one half of one paper towel was provided as nesting material (Fig. 1). Pups were randomly distributed to dams until each litter contained 12 pups with equal male: female ratio. These cages then remained undisturbed for 7 days. On PND 10, all litters were transferred to and maintained in standard cages until offspring were weaned on PND 21. On PND 10, 21 and 35, equal numbers of offspring were anesthetized with isoflurane and euthanized by decapitation. The bilateral tibia and lumbar vertebra were collected and stored at − 80 °C until analyses.

### Bone Analyses

Bones were cleaned of adhering muscle tissues. Length of the tibia was measured using a digital caliper (Sheffield, England). Tibial and vertebral bone mineral area (BMA), content (BMC), and density (BMD) were assessed using dual X-ray absorptiometry (DEXA; Lunar PIXI with PIXImus Series Software version 1.4X, GE Medical Systems, Madison, WI). A quality control phantom mouse was scanned before sample measurements were taken to assure no more than 2% deviations were recorded in measurements.

Bone microarchitecture of the tibia and 4th lumbar vertebrae were assessed using microcomputed tomography (μCT40, SCANCO Medical, Switzerland). For the tibia, only the cortical bone was analyzed at the mid-diaphysis, as the growth plate was not visible in these young, growing rats, excluding trabecular analysis. The midshaft of the tibia was evaluated by acquiring 50 slices at the midpoint and analyzing 30 slices (180 μm). Tibial cortical bone parameters that were assessed included the bone volume expressed as a percentage of the total volume (BV/TV), cortical thickness (CoTh), cortical area (CA), bone perimeter (B.Pm), and medullary area.

Vertebral samples were analyzed by acquiring images at a resolution of 1024 × 1024 pixels, ~ 30 μm from the dorsal and caudal growth plates. At the proximal metaphysis, semi-automated contours were placed to include the secondary spongiosa within the volume of interest (VOI) that included 160 to 180 slices. All analyses were performed at a threshold of 250–280 and a sigma and support of 1.2 and 2, respectively. The vertebral trabecular bone parameters assessed included BV/TV, connectivity density (Conn.D), structural model index (SMI), trabecular thickness (Tb.Th), trabecular number (Tb.N), trabecular separation (Tb.Sp), material density (MD), and degree of anisotropy (DA).

### RNA Extraction, Library Preparation, and Gene Annotation

The vertebrae were homogenized using TRIzol reagent (Sigma-Aldrich, St. Louis, MO), and the homogenate was separated into aqueous and organic phases by adding 1-bromo-3-chloropropane (BCP) (Millipore Sigma) and centrifugation. Total RNA was obtained from the aqueous phase using a miRNeasy kit (Qiagen, Germantown, MD) following the manufacturer’s standard protocol. RNA concentration was obtained by measuring optical density at 260 nm wavelength and purity with 260/280 ratio (1.9 to 2.1) using Nanodrop spectrophotometer (Wilmington, DE). RNA quality was determined using RNA Screen Tape Station and analyzed with software A.02.02-SR1 (Agilent Technologies, Inc, Santa Clara, CA). Samples with RNA integrity numbers of 5.5–9.6. RNA were shipped to Novogene Co. Ltd. (USA) for library preparation and sequencing. mRNA was purified from total RNA using poly-T oligo-attached magnetic beads, and stranded mRNA-seq libraries were generated and individually barcoded using NEBNext® Ultra Directional RNA Library Prep Kit for Illumina (NEB, USA) following manufacturer’s recommendations. Fragmentation was carried out using divalent cations under elevated temperature in NEB Next First-Strand Synthesis Reaction Buffer (5X). First-strand cDNA was synthesized using random hexamer primer and M-MuLV Reverse Transcriptase (RNase H-) ring followed by the second-strand synthesis in the NEBNext Ultra II Directional RNA Library prep protocol, the reaction uses a buffer containing dUTP, which allows the incorporation of dUTP in place of dTTP. This process is facilitated by the NEBNext Second-Strand Synthesis Reaction Buffer with dUTP mix and the NEBNext Second-Strand Synthesis Enzyme Mix. The incorporation of dUTP is essential for strand specificity, as it renders the second-strand cDNA uracil-containing, making it susceptible to degradation during subsequent PCR steps. This selective degradation ensures that only the first-strand cDNA is amplified, preserving strand orientation in the final library. Remaining overhangs were converted into blunt ends via exonuclease/polymerase activities. After adenylation of 3’ ends of DNA fragments, NEBNext Adaptor with hairpin loop structure was ligated to prepare for hybridization. To select cDNA fragments of preferentially 150 ~ 200 bp in length, the library fragments were purified with AMPure XP system (Beckman Coulter, Beverly, USA). Then, 3 µl USER Enzyme (NEB, USA) was used with size-selected, adaptor ligated cDNA at 37 °C for 15 min followed by 5 min at 95 °C before PCR. PCR was performed with Phusion High-Fidelity DNA polymerase, Universal PCR primers, and Index Primer, and PCR products were purified (AMPure XP system). Library quality was assessed on the Agilent Bioanalyzer 2100 system and sequenced by NovaSeq6000 on an S4 flow cell (PE150 read length).

Quality filtered and trimmed raw fastq files were aligned to the rat genome using STAR [35, 36] aligner (version 2.7.9a) and counted by Feature Counts (v.1.5.0-p3) [24]. Differential expression was performed using DESeq2 in R 4.2.0 [37], and the resulting differentially expressed genes (DEGs) were determined using the design matrix of the different factors, such as timepoints, condition and sex, at a defined cut-off criteria of |log_2_ fold-change|≥ 0.25 and *p* value < 0.1 adjusted using the Benjamini and Hochberg’s approach to control the false discovery rate. DEGs were subjected to REACTOME pathway analyses to determine the significantly enriched pathways. To extract further biological insight from the samples, we implemented gene set enrichment analysis (GSEA) [38] which assesses the statistical enrichment of gene ontologies, and pathways, at a 5% significance, and data were visualized using Cluster profiler v4.8.1 [39].

### Statistical Analyses

Statistical analysis of bone parameters was conducted using the Prism software (GraphPad software; version 9.5; Boston, MA). Data were analyzed using 2 factor (factors: stress and sex) analysis of variance (2-way ANOVA) using the mixed model procedure, followed by Tukey’s post hoc test.

## Results

### CES Impairs Growth Rate During the Lactation But Not In Post Weaning Period

**Fig. 2:**
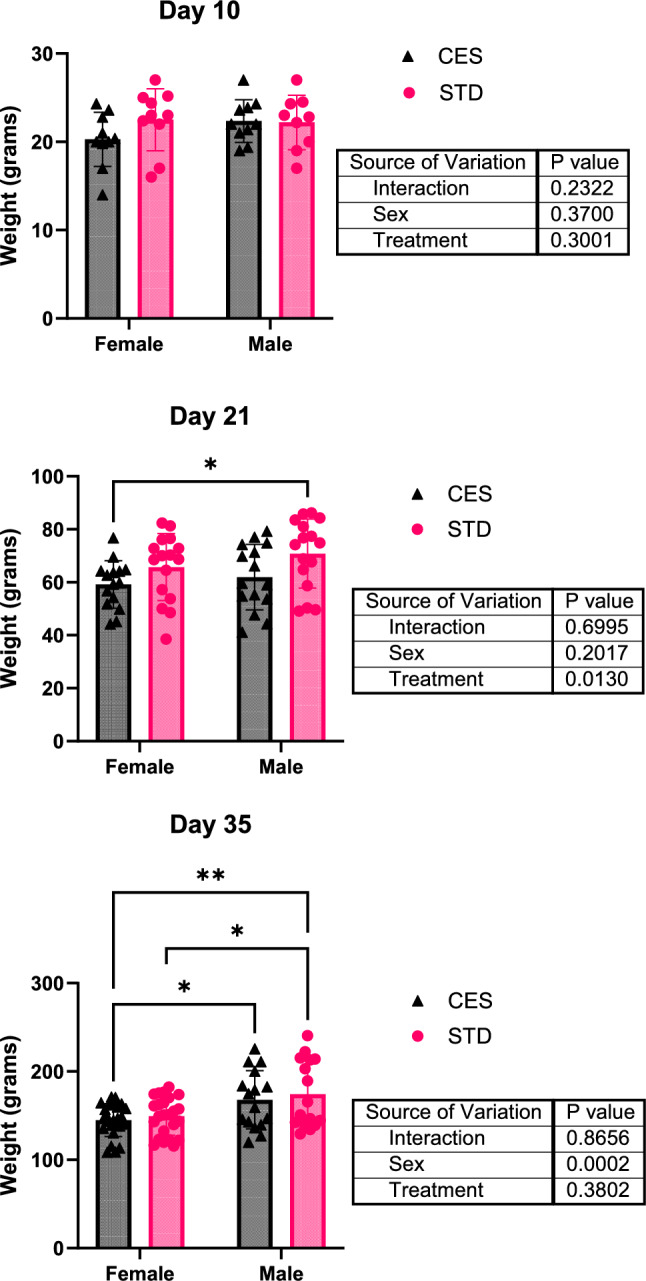
Effect of CES on body weight. Average weight of rats in the control and chronic early stress groups at PND 10, 21, 35. Data are presented as mean ± SD from *n* = 10–25/sex/group/postnatal day. Two-way ANOVA and Tukey’s test with *p* < 0.05 were considered significant. STD—control; chronic early-life stress—CES; * = *p* < 0.05

To evaluate whether CES affected general growth, body weights were measured across developmental time points. At PND 10 and PND 35, no significant differences in body weight were observed between CES and STD groups. However, at PND 21, offspring in the CES group had significantly lower body weight (*p* < 0.01), indicating a temporary growth delay potentially due to maternal stress or limited nutrition during the lactation period. (Fig. 2).

### Tibial Bone Mineral Content and Length Decreased in Response to CES Across Developmental Stages

At PND 10, tibial BMD and BMC showed no significant differences between treatment groups or sex (Table 1). However, tibial BMA (*p* = 0.0006) and tibial length (*p* = 0.032) were lower in the CES group. A significant treatment–sex interaction (*p* = 0.036) was observed with tibial length of CES males decreasing tibial length but not in the females. By PND 21, CES showed a trend toward lower tibial BMD (*p* < 0.078) and significantly lower BMA (*p* = 0.021), BMC (*p* = 0.001), and length **(***p* < 0.0001) compared to STD. At PND 35, neither BMD nor BMC showed significant treatment or sex differences (Table 1). However, tibial length was shorter (*p* = 0.05) in the CES group, suggesting that CES continues to influence bone growth**.**

**Table 1:** Effect of CES on tibial length and densitometry parameters at postnatal day (PND) 10, 21, and 35

Parameter	STD Male	CES Male	STD Female	CES Female	P value		
					Treatment	Sex	Interaction
PND 10							
BMD (mg/cm^2^)	22.92 ± 1.47	22.85 ± 2.25	23.33 ± 1.93	22.30 ± 2.26	0.439	0.923	0.503
BMC (mg)	7.37 ± 7.55	4.37 ± 0.74	5.00 ± 0.00	4.00 ± 0.53	0.148	0.315	0.463
BMA (cm^2^)	0.21 ± 0.02	0.18 ± 0.03	0.21 ± 0.01	0.17 ± 0.02	0.0006	0.768	0.462
Length (cm)	1.42 ± 0.20^a^	1.19 ± 0.17^b^	1.22 ± 0.05^b^	1.21 ± 0.09^b^	0.0324	0.086	0.036
PND 21							
BMD (mg/cm^2^)	47.05 ± 5.04	45.15 ± 4.08	48.55 ± 4.93	45.19 ± 4.90	0.078	0.597	0.616
BMC (mg)	47.55 ± 4.76	42.20 ± 4.54	47.36 ± 6.19	39.64 ± 8.26	0.001	0.469	0.529
BMA (cm^2^)	1.02 ± 0.13	0.94 ± 0.09	0.97 ± 0.12	0.87 ± 0.13	0.021	0.168	0.758
Length (cm)	2.47 ± 0.10	2.37 ± 0.06	2.47 ± 0.11	2.29 ± 0.11	< 0.0001	0.221	0.191
PND 35							
BMD (mg/cm^2^)	92.91 ± 6.28	90.50 ± 4.16	92.38 ± 8.35	89.70 ± 9.02	0.268	0.769	0.954
BMC (mg)	127.95 ± 48.42	140.40 ± 17.5	144.40 ± 19.4	137.20 ± 20.5	0.738	0.396	0.211
BMA (cm^2^)	1.55 ± 0.19	1.55 ± 0.19	1.56 ± 0.20	1.53 ± 0.19	0.772	0.995	0.746
Length (cm)	3.16 ± 0.14	3.13 ± 0.13	3.28 ± 0.22	3.11 ± 0.14	0.048	0.281	0.181

Values are expressed as mean ± standard deviation (SD). PND 10: Male STD (*n* = 8), Male CES (*n* = 8), Female STD (*n* = 8), Female CES (*n* = 8). PND 21: Male STD (*n* = 11), Male CES (*n* = 10), Female STD (*n* = 11), Female CES (*n* = 11). PND 35: Male STD (*n* = 8), Male CES (*n* = 10), Female STD (*n* = 15), Female CES (*n* = 16). STD- control; CES—chronic early-life stress; BMD—Bone mineral density; BMC—Bone mineral content; BMA—Bone mineral area. Statistical analysis was conducted using two-way ANOVA with Tukey’s post hoc test and significance is defined as *p* < 0.05

### CES Reduces Tibial Mid-Diaphysis Bone Volume and Alters Microarchitecture Across Developmental Stages

As shown in Table 2, at PND 10, no significant effects of CES or sex were observed for most cortical bone parameters, including total volume (TV), cortical area (CA), and medullary area (MA). The exception was cortical thickness, which was significantly higher in the CES group (*p* < 0.0001), suggesting an early impact of CES on this microstructural feature. Interestingly, bone perimeter (B.Pm; *p* < 0.056) tended to be lower in CES groups than in control offspring. By PND 21, CES resulted in significant reductions in TV (*p* = 0.036) and CA (*p* < 0.017), indicating that CES impacts the cortical bone parameters in weaned offspring (Table 2). Notably, cortical thickness did not differ significantly at this stage, suggesting that the initial increase observed at PND 10 did not persist. At PND 35, no CES effects were observed but sex differences emerged, with significant changes in TV (*p* = 0.048) and CA (*p* = 0.002), with males displaying higher values compared to females (Table 2). These findings suggest that CES has a lasting impact on certain aspects of cortical bone microarchitecture, particularly concerning total volume, with sex-specific differences becoming more pronounced as the animals mature.

**Table 2:** Effect of chronic early-life stress (CES) on tibial mid-diaphysis microcomputed tomography parameters at postnatal day (PND) 10, 21, and 35

Parameter	STD Male	CES Male	STD Female	CES Female	P value		
					Treatment	Sex	Interaction
PND 10							
B.Pm (mm)	18.35 ± 2.99	17.14 ± 3.17	17.90 ± 1.26	15.71 ± 1.67	0.056	0.280	0.574
Co.Th (mm)	0.04 ± 0.01	0.05 ± 0.006	0.04 ± 0.00	0.05 ± 0.004	< 0.0001	0.355	0.086
CA (mm^2^)	0.37 ± 0.07	0.38 ± 0.07	0.35 ± 0.03	0.37 ± 0.03	0.362	0.396	0.689
MA (mm^2^)	0.29 ± 0.06	0.29 ± 0.19	0.27 ± 0.06	0.22 ± 0.07	0.510	0.235	0.538
TV (mm^3)	0.10 ± 0.02	0.10 ± 0.04	0.10 ± 0.01	0.09 ± 0.01	0.886	0.221	0.755
PND 21							
B.Pm (mm)	15.16 ± 1.99	14.67 ± 1.21	14.64 ± 2.61	13.89 ± 1.35	0.299	0.273	0.825
Co.Th (mm)	0.16 ± 0.03	0.16 ± 0.02	0.18 ± 0.04	0.17 ± 0.03	0.512	0.233	0.705
CA (mm^2^)	1.15 ± 0.09	1.11 ± 0.13	1.18 ± 0.09	1.07 ± 0.08	0.017	0.775	0.239
MA (mm^2^)	0.17 ± 0.23	0.12 ± 0.04	0.11 ± 0.05	0.10 ± 0.03	0.411	0.293	0.607
TV (mm^3)	0.21 ± 0.04	0.19 ± 0.02	0.20 ± 0.01	0.18 ± 0.01	0.036	0.336	0.336
PND 35							
B.Pm (mm)	13.90 ± 2.18	14.30 ± 0.97	13.67 ± 1.21	13.71 ± 1.49	0.608	0.347	0.671
Co.Th (mm)	0.36 ± 0.05	0.35 ± 0.04	0.34 ± 0.05	0.33 ± 0.04	0.751	0.158	0.845
CA (mm^2^)	2.38 ± 0.12	2.37 ± 0.23	2.21 ± 0.13	2.20 ± 0.21	0.879	0.002	0.926
MA (mm^2^)	0.13 ± 0.17	0.12 ± 0.15	0.17 ± 0.39	0.07 ± 0.02	0.482	0.925	0.527
TV (mm^3)	0.39 ± 0.01	0.39 ± 0.04	0.37 ± 0.07	0.35 ± 0.03	0.498	0.048	0.638

Values are expressed as mean ± standard deviation (SD). PND 10: Male STD (*n* = 8), Male CES (*n* = 8), Female STD (*n* = 8), Female CES (*n* = 8). PND 21: Male STD (*n* = 11), Male CES (*n* = 10), Female STD (*n* = 11), Female CES (*n* = 11). PND 35: Male STD (*n* = 8), Male CES (*n* = 10), Female STD (*n* = 15), Female CES (*n* = 16). STD- Control; CES—chronic early-life stress; B.Pm- Bone perimeter; Co.Th- Cortical thickness; CA—Cortical area; MA—Medullary area; TV- Total volume. Statistical analysis was conducted using two-way ANOVA with Tukey’s post hoc test and significance is defined as *p* < 0.05

### CES Impairs L4 Vertebral Bone Density and Content, with Pronounced Effects in Female Offspring Across Developmental Stages

**Fig. 3:**
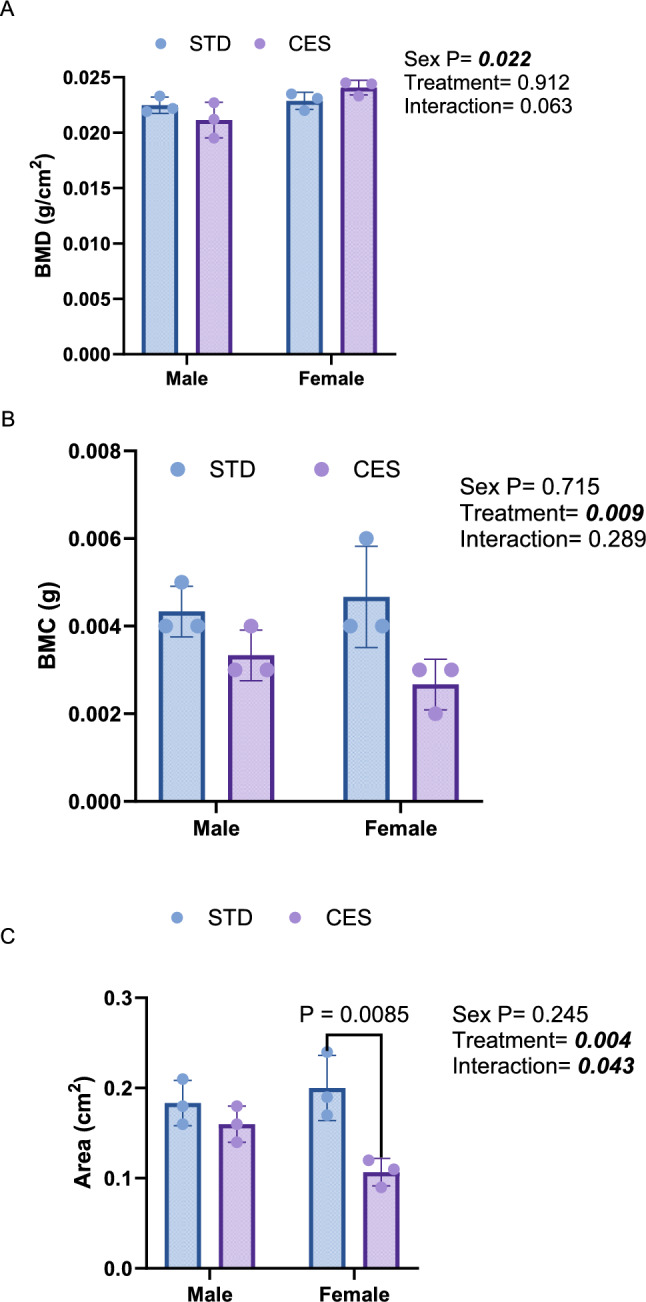
Effects of CES on lumbar bone densitometric parameters at postnatal day (PND) 10. **A** bone mineral density, BMD, **B** bone mineral content, BMC, and **C** bone mineral area, BMA, Data are presented as mean ± SD from *n* = 3/sex/group. Two-way ANOVA and Tukey's test with *p* < 0.05 were considered significant. STD—control; chronic early-life stress—CES

**Fig. 4:**
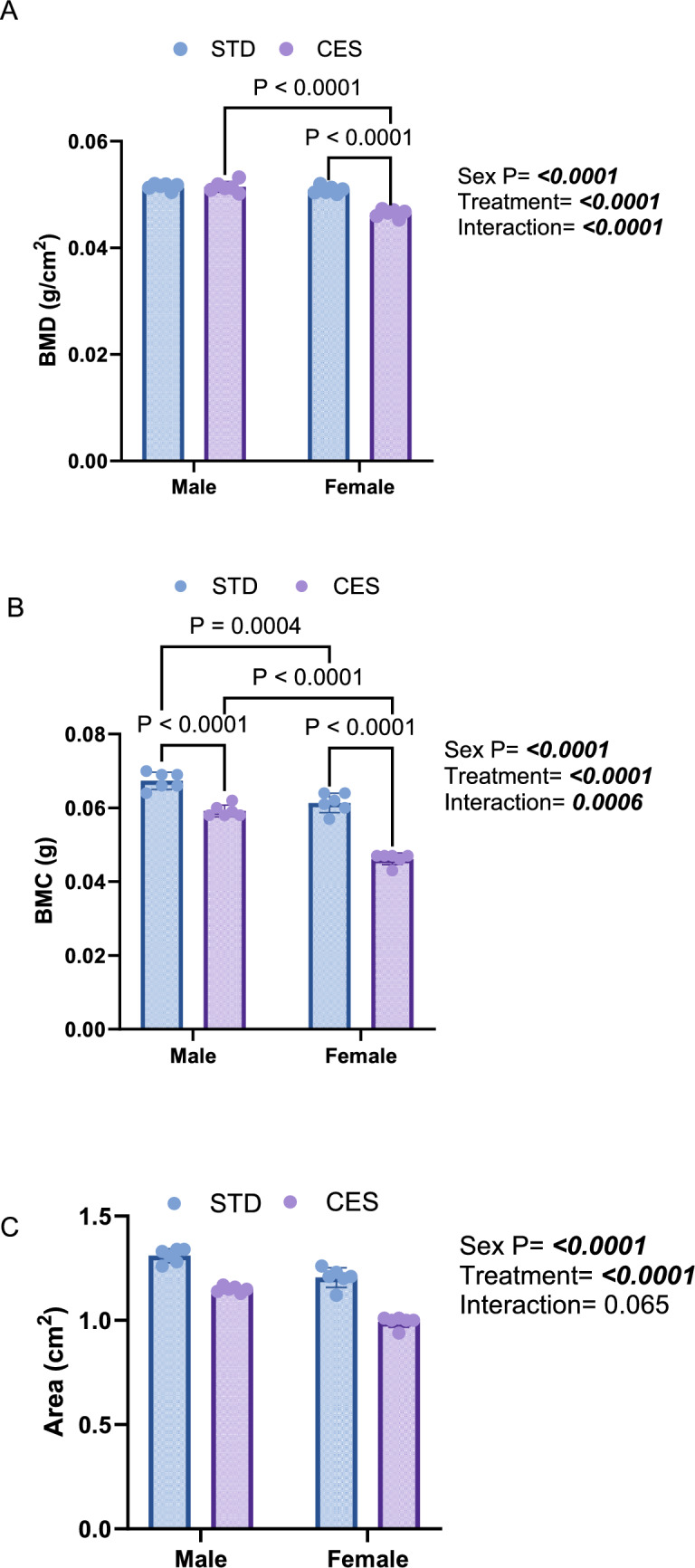
Effects of CES on lumbar bone densitometric parameters at postnatal day (PND) 21. **A** bone mineral density, BMD, (**B**) bone mineral content, BMC, and **C** bone mineral area, BMA. Data are presented as mean ± SD from *n* = 6/sex/group. Two-way ANOVA and Tukey's test with *p* < 0.05 were considered as significant. STD—control; chronic early-life stress—CES

**Fig. 5:**
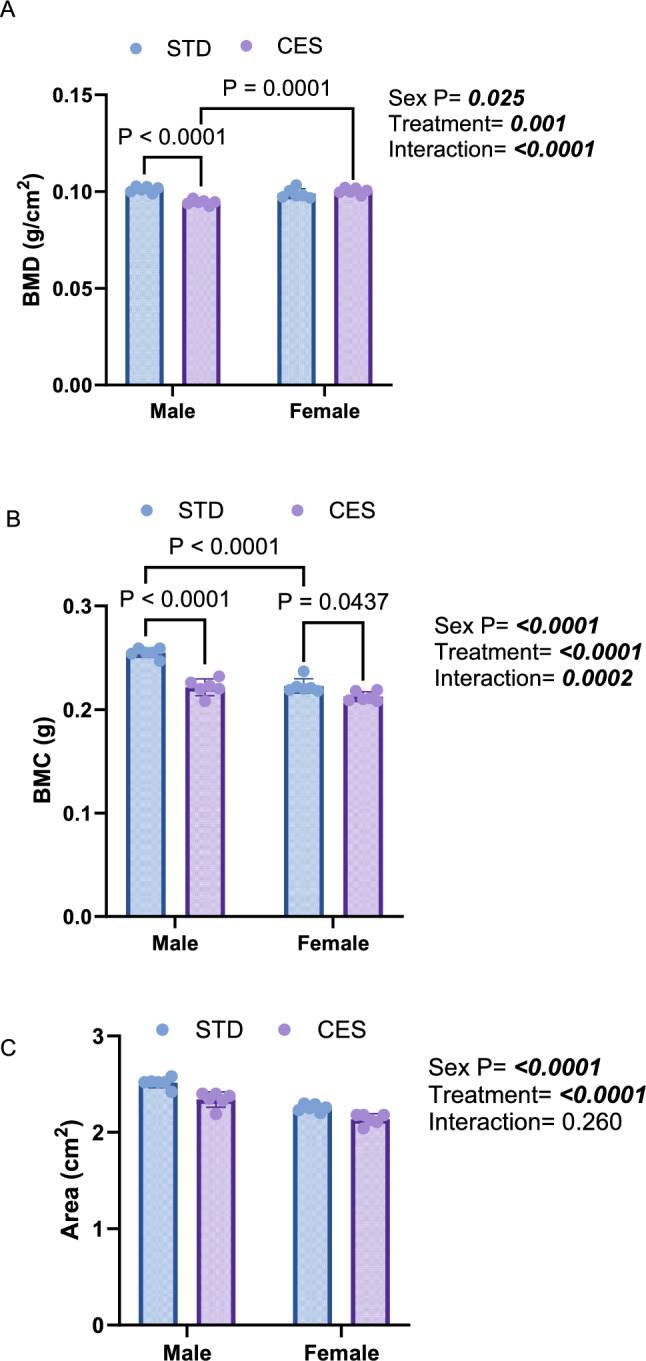
Effects of CES on lumbar bone densitometric parameters at postnatal day (PND) 35. **A** bone mineral density, BMD, **B** bone mineral content, BMC, and **C** bone mineral area, BMA. Data are presented as mean ± SD from *n* = 6/sex/group. Two-way ANOVA and Tukey's test with *p* < 0.05 were considered as significant. STD—control; chronic early-life stress—CES

In the lumbar vertebra, BMD at PND 10 did not exhibit significant treatment effects but showed a significant sex effect (*p* = 0.02) with higher BMD in males compared to females and a marginal interaction effect (*p* = 0.06; Fig. 3A). CES caused a significant reduction in BMC (*p* = 0.009; Fig. 3B) as well as in BMA (*p* = 0.004) in CES, particularly in females (interaction effect, *p* = 0.044; Fig. 3C). At PND 21, BMD was significantly lower in the CES group compared to the control group (*p* < 0.0001) and was also significantly lower in females compared to males (*p* < 0.0001), with a significant interaction effect (*p* < 0.0001; Fig. 4A**)**. BMC also significantly decreased due to CES (*p* < 0.0001), influenced by sex (*p* < 0.0001) with a significant interaction effect (*p* = 0.0006 Fig. 4B). BMA was significantly lower (*p* < 0.0001) in the CES group and in females **(**Fig. 4C). By PND 35, BMD remained significantly lower in the CES group (*p* = 0.002), with significant sex (*p* = 0.026) and interaction (*p* < 0.0001; Fig. 5A) effects. Similarly, BMC showed significant reductions due to treatment and sex effects, with a significant interaction (all *p* < 0.0001; Fig. 5B). BMA was also significantly reduced in the CES group (*p* < 0.0001), with significant sex effects (*p* < 0.0001) and no significant interaction effect (*p* = 0.261; Fig. 5C). These results demonstrate that CES adversely affects vertebral bone density, especially in females, throughout different developmental stages.

### CES Alters L4 Vertebral Trabecular Structure and Anisotropy of the Offspring with Sex-Specific Effects at Later Stages

No group differences were observed in the morphometric parameters Tb.Th, Tb.N, or Tb.Sp due to CES at PND 21 and 35 (Table 3). However, at PND 21, BV/TV showed a significant sex effect (*p* < 0.0001), with females exhibiting higher values compared to males, but there were no treatment or interaction effects. Material density was also significantly influenced by sex (*p* < 0.0001), with females exhibiting lower MD compared to males, but there were no treatment or interaction effects. Additionally, the DA, which reflects the directional dependence of trabecular organization, showed a significant treatment effect (*p* = 0.04), with higher values in the CES group. This suggests that CES influences trabecular alignment, although sex and interaction effects were not significant. For SMI, males exhibited significantly higher values (more rod-like trabecular structure) compared to females (*p* < 0.0001), but there were no treatment or interaction effects (Table 3). By PND 35, BV/TV tended to be lower in the CES group (*p* = 0.054), with a significant sex effect (*p* = 0.005), where males exhibited lower BV/TV than females (Table 3). SMI was significantly higher in the CES group (*p* = 0.048) and among males (*p* = 0.001), indicating a more rod-like trabecular structure, particularly in stressed male rats. Furthermore, the DA exhibited a significant interaction effect (*p* = 0.046), further emphasizing the impact of CES on the orientation of the trabecular structure (Table 3).

**Table 3:** Effect of chronic early-life stress (CES) on lumbar vertebra microcomputed tomography parameters at postnatal day (PND) 21 and 35

Parameter	STD Male	CES Male	STD Female	CES Female	P value		
					Treatment	Sex	Interaction
PND 21							
BV/TV	0.28 ± 0.03	0.28 ± 0.01	0.35 ± 0.03	0.35 ± 0.03	0.962	< 0.0001	0.820
Tb.Th (mm)	0.10 ± 0.02	0.13 ± 0.01	0.13 ± 0.01	0.13 ± 0.02	0.084	0.065	0.054
Tb.N (mm)	3.53 ± 0.81	2.83 ± 0.23	2.87 ± 0.44	3.13 ± 0.83	0.445	0.537	0.105
Tb.Sp (mm)	0.29 ± 0.05	0.33 ± 0.05	0.32 ± 0.05	0.31 ± 0.07	0.613	0.818	0.214
MD (mgHA/cm^3^)	746.39 ± 4.68	778.87 ± 7.02	730.08 ± 6.08	732.84 ± 18.06	0.073	< 0.0001	0.2119
Conn.D (1/mm^3^)	92.11 ± 11.70	87.28 ± 11.43	81.99 ± 11.59	87.70 ± 14.24	0.344	0.383	0.936
DA	1.21 ± 0.03	1.24 ± 0.06	1.23 ± 0.04	1.28 ± 0.03	0.041	0.071	0.602
SMI	1.07 ± 0.18	1.11 ± 0.10	0.23 ± 0.44	0.31 ± 0.56	0.722	< 0.0001	0.918
PND 35							
BV/TV	0.35 ± 0.02	0.32 ± 0.02	0.39 ± 0.04	0.36 ± 0.04	0.054	0.005	0.971
Tb.Th (mm)	0.11 ± 0.02	0.11 ± 0.01	0.12 ± 0.01	0.12 ± 0.00	0.763	0.152	0.689
Tb.N (mm)	3.29 ± 0.59	3.04 ± 0.49	3.26 ± 0.90	3.04 ± 0.30	0.383	0.958	0.964
Tb.Sp (mm)	0.30 ± 0.05	0.31 ± 0.04	0.29 ± 0.06	0.30 ± 0.03	0.653	0.661	0.891
MD (mgHA/cm^3^)	847.70 ± 10.76	845.98 ± 16.75	845.37 ± 14.42	845.41 ± 10.51	0.538	0.607	0.369
Conn.D (1/mm^3^)	80.02 ± 10.05	80.36 ± 6.51	93.61 ± 23.86	82.51 ± 6.34	0.387	0.211	0.358
DA	1.44 ± 0.12^a^	1.33 ± 0.04^a^	1.38 ± 0.12^a^	1.45 ± 0.07^a^	0.648	0.434	0.046
SMI	0.38 ± 0.22	0.73 ± 0.24	-0.05 ± 0.35	0.18 ± 0.42	0.048	0.001	0.659

Values are expressed as mean ± standard deviation (SD) rom n = 6 for all groups.

STD—control; CES—chronic early-life stress; BV/TV—Bone volume to total volume ratio; Tb.Th—Trabecular thickness; Tb.N—Trabecular number; Tb.Sp—Trabecular separation; MD—Material density; Conn.D—connectivity density; DA—Degree of anisotropy; SMI—Structure model index. Statistical analysis was conducted using two-way ANOVA with Tukey’s

### CES Impacted Gene Expression of 4th Lumbar Vertebrae in the Offspring

**Fig. 6:**
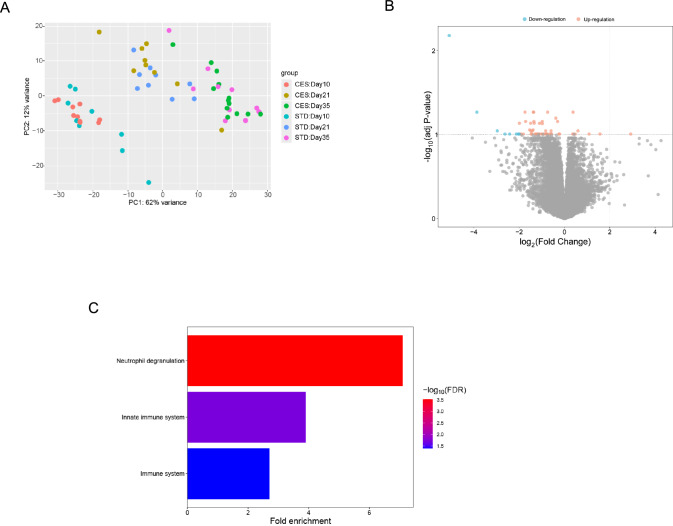
Influence of CES on spine (Lumbar vertebrae 4) gene expression profile in PND21 rat offspring. The principal component analysis (PCA) plot shows no group (STD and CES) clustering across three different time points **A**. An overview of differentially expressed genes between the CES and STD group at day 21 **B**. Bar graph showing the list of significantly enriched pathways in the STD group compared to the CES group on day 21 **C**. DEGs—differentially expressed genes; STD—standard; CES—chronic early-life stress. Sample *n* = 9/group with 4 males and 5 females in each group

To investigate the overall impact of CES on the transcriptome profile of the bone lumbar vertebrae, we conducted mRNA-seq at PND 10, 21, and 35. PCA showed no clear separation between males and females (data not shown); therefore, we combined the data for males and females for further analysis (Fig. 6A). Differential gene expression analysis (DEG) revealed no difference at PND 10 and 35 between CES and STD groups. Interestingly, at PND21, we observed 66 DEGs (log_2_ fold-change > 0.25 and adj *p* value < 0.1; Fig. 6B, Supplemental table 1) between the CES and STD groups, of which 57 and 9 were downregulated and -upregulated, respectively, in the CES compared to the STD group. We performed REACTOME pathway enrichment analysis to understand the functional significance of these DEGs. We observed that downregulated genes in the CES group were significantly enriched in neutrophil degranulation and innate immune and immune system pathways (False discovery rate, FDR, *p* value < 0.05; Fig. 6C).

**Fig. 7:**
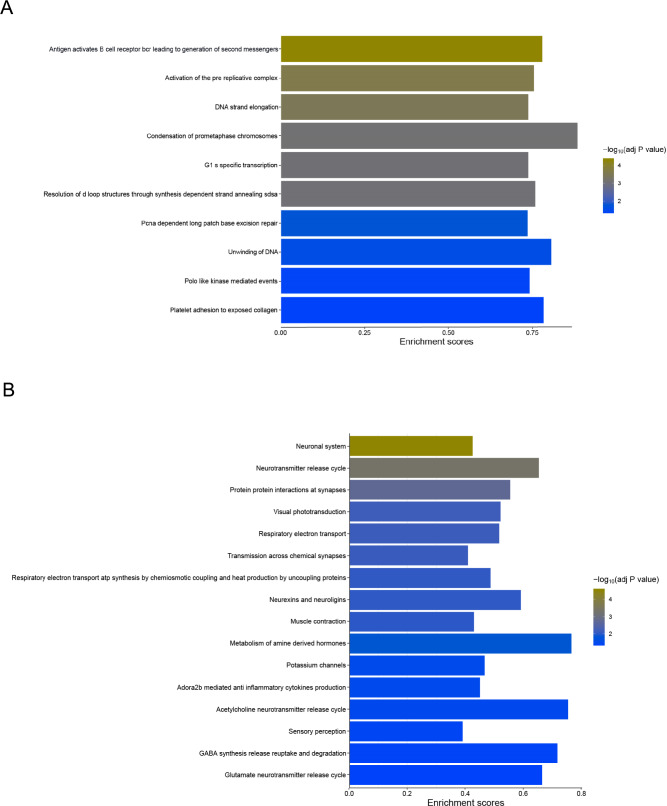
Gene set enrichment analysis. The top significantly enriched REACTOME pathways in STD **A** and CES **B** groups at PND 21. STD—standard; CES—chronic early-life stress. Sample *n* = 9/group with 4 males and 5 females in each group

To capture the subtle molecular differences between the groups fully, as some individual genes may not meet the statistical threshold after adjusting for multiple *p* value correction, we performed Gene Set Enrichment Analysis (GSEA) using the whole transcriptome. No pathways were enriched in vertebral bone of the STD or CES group at PND 10 and 35. However, at PND 21, 135 pathways were significantly enriched in the vertebral bone of the STD group, while only 17 pathways were significantly enriched in the CES group. The top 10 significantly activated pathways in the STD group were involved in cell growth and development-related pathways, including condensation of prometaphase chromosomes, the unwinding of DNA, activation of the pre-replicative complex, polo-like kinase-mediated events, DNA strand elongation, and G1 specific transcription (Fig. 7A), whereas the significantly enriched pathways in the CES group were involved in neurotransmission signaling-related pathways, such as transmission across chemical synapses, neuronal system neurotransmitter release cycle, glutamate neurotransmitter release cycle, GABA synthesis release reuptake and degradation, acetylcholine neurotransmitter release cycle, protein–protein interactions at synapses and neurexins and neuroligins (Fig. 7B). The full list of significantly enriched pathways in the STD and CES group is found in Supplemental table 2.

## Discussion

Limited resources during early life, including both prenatal and postnatal periods, have been shown to significantly affect the offspring by impairing cognitive development, increasing susceptibility to immune dysfunction, and reducing BMD [5, 40, 41]. CES disrupts various physiological systems, including skeletal development [42, 43]. However, most CES-related bone studies have focused on aging populations [44–47] or utilized maternal separation models [43]. In contrast, our study employed the LBN model, a minimally invasive paradigm designed to mimic resource scarcity while preserving maternal presence, to evaluate postnatal skeletal development. Compared to maternal separation, LBN may more accurately simulate limited resources in early life [43]. The postnatal timepoints selected, PND 10, 21, and 35, correspond to early infancy, childhood, and preadolescence, respectively, in humans [48], providing a translational framework for developmental evaluation. Our findings support the hypothesis that limited resources act as a significant risk factor for altered bone growth and mineralization.

Tibial BMC, indicative of calcium and phosphorus accumulation, was significantly reduced by CES at PND 21 but not at PND 10 or 35, suggesting a specific vulnerability during this window of development. Interestingly, tibial length was consistently reduced across all ages in CES rats, suggesting potential growth retardation. Since the mother is the primary source of nourishment prior to weaning, it is possible that altered maternal care or milk composition contributed to reduced mineral accrual [49, 50]. However, this remains speculative and warrants further investigation in future studies.

The observed BMD reduction is consistent with published findings on the long-term skeletal consequences of early-life stress [42, 43, 51–53]. For instance, maternal separation in rodents resulted in reduced BMD at 8–10 months of age [43], while stress-induced models, such as electric foot shock or PTSD induction, similarly showed BMC/BMD loss in prepubertal mice but not in adults [51]. Importantly, Zupan et al. [54] linked these changes to increased osteoclastogenesis, suggesting stress-driven enhancement of bone resorption pathways. Microarchitectural assessments revealed early effects of CES on cortical bone. At PND 10, CES increased cortical thickness, potentially suggesting compensatory mineral deposition; however, this was coupled with lower bone perimeter, indicating reduced bone size and possibly diminished structural integrity. By PND 21, CES significantly decreased cortical total volume and area, markers of compromised growth. These effects were no longer significant by PND 35, though sex differences emerged, with males showing greater cortical area and volume. These findings highlight the age-specific nature of the impacts of CES with limited resources setting and suggest partial recovery or adaptation after weaning.

Notably, CES caused persistent reductions in vertebral BMC and BMD, particularly in females, across all time points. Given the high trabecular content of the lumbar vertebrae and its metabolic sensitivity, these findings suggest long-term susceptibility of axial skeleton to CES. Increased DA at PND 21 and elevated SMI at PND 35 further suggest altered bone architecture and a shift toward mechanically unfavorable, rod-like trabecular structures. Such changes may reduce peak bone mass accrual and increase fracture risk, emphasizing the clinical relevance of preventing CES.

In terms of molecular mechanisms, gene expression analysis of the L4 vertebra at PND 21 provided insight into stress-induced alterations. We chose the L4 vertebra for transcriptomic analysis due to observed structural and mineralization changes, as well as the role of bone as a sensitive target of stress-regulated hormonal and metabolic pathways. Though RNA-seq in bone presents challenges due to tissue heterogeneity, recent literature supports its utility in identifying relevant biological changes when interpreted in the context of supporting phenotypic data [39]. Our data revealed upregulation of HOXA4 and FOS—genes associated with inhibited cell proliferation and disrupted bone remodeling, respectively [55]. Moreover, we identified downregulation of neutrophil degranulation pathways, which may suggest suppressed immune activity or altered bone marrow niche function. While previous work showed similar suppression in response to physical stress (e.g., exercise) [56–58], our findings extend this to early-life limited resources paradigm. Gene set enrichment analysis showed that in standard (STD) conditions, pathways related to cell growth and differentiation were dominant, consistent with increased BMC and BMD. In contrast, CES animals exhibited enrichment of neurotransmitter-related pathways (e.g., glutamate, GABA, acetylcholine release cycles), aligning with previous studies showing that chronic stress perturbs central neurotransmitter systems [52, 59, 60]. Though speculative, the presence of such pathways in bone may reflect peripheral neural-immune interactions or stress hormone signaling, which are known to influence bone remodeling.

Our study has several limitations. First, this is a descriptive study lacking histological, bone turnover, and mechanical strength assessments. These analyses would provide deeper insights into bone cell activity, osteoid formation, and structural resilience, and should be prioritized in future work. Second, shorter bones could reflect developmental delay. Although litter size and survival were similar between groups, body weights were lower in CES pups at PND 21 but normalized by PND 35 (Fig. 2), supporting a transient growth delay rather than a permanent deficit with limited resources paradigm. We did not assess maternal milk production, pup suckling behavior, or locomotion, which are relevant to bone development. However, our goal was to minimize pup handling to avoid confounding stressors during the sensitive LBN period. No observable differences in stomach milk content weights were noted at PND 10 (data not shown). Furthermore, while cross-fostering experiments could help distinguish maternal versus direct pup effects, this was beyond the scope of the current study. We have discussed this as a critical future direction and recognize it as a limitation. Lastly, while bulk RNA-seq lacks cellular resolution, our gene expression changes correspond with phenotypic differences, particularly at PND 21 when bone and body weight changes were also observed, the current transcriptomic data provide an informative molecular snapshot of CES-induced disruption in bone development.

In conclusion, our study provides evidence that early-life CES through limited bedding and nesting alters bone growth, mineralization, and structure during critical developmental windows. These effects appear age- and sex-specific, with persistent vertebral deficits and transient cortical changes. Molecular analyses suggest disrupted growth and immune signaling. Given the potential for long-term skeletal consequences, interventions aimed at maternal support and stress mitigation may be key to improving developmental outcomes in the offspring.

## Supplementary Information

Below is the link to the electronic supplementary material.

Supplementary Table 1. The list of differentially expressed genes in lumbar vertebrae of chronic early life stress (CES) and control (STD) group in PND 21. log2FoldChange - log fold-change of the average expression between the two groups (positive and negative values indicate high and low expression of that gene, respectively, in CES group compared to the STD group). Sample n = 9/group with 4 males and 5 females in each group. Supplementary file1 (XLSX 11 kb)

Supplementary Table 2. List of REACTOME pathways that were significantly enriched in PND 21 rat spine (lumbar vertebrae 4) of CES and STD group as identified by gene set enrichment analysis. Sample n = 9/group with 4 males and 5 females in each group. Supplementary file2 (XLSX 17 kb)

## Data Availability

Transcriptome data are available at https://www.ncbi.nlm.nih.gov/geo/query/acc.cgi?acc=GSE297538.
